# All urban areas’ energy use data across 640 districts in India for the year 2011

**DOI:** 10.1038/s41597-021-00853-7

**Published:** 2021-04-12

**Authors:** Kangkang Tong, Ajay Singh Nagpure, Anu Ramaswami

**Affiliations:** 1 Department of Civil and Environmental Engineering, Princeton University, Princeton, NJ USA; 2 Hubert H. Humphrey School of Public Affairs, University of Minnesota Twin Cities, Minneapolis, MN USA; 3 M.S. Chadha Center for Global India, Princeton University, Princeton, NJ USA; 4 High Meadows Environmental Institute, Princeton University, Princeton, NJ USA

**Keywords:** Environmental impact, Environmental sciences

## Abstract

India is the third-largest contributor to global energy-use and anthropogenic carbon emissions. India’s urban energy transitions are critical to meet its climate goals due to the country’s rapid urbanization. However, no baseline urban energy-use dataset covers all Indian urban districts in ways that align with national totals and integrate social-economic-infrastructural attributes to inform such transitions. This paper develops a novel bottom-up plus top-down approach, comprehensively integrating multiple field surveys and utilizing machine learning, to model All Urban areas’ Energy-use (AllUrE) across all 640 districts in India, merged with social-economic-infrastructural data. Energy use estimates in this AllUrE-India dataset are evaluated by comparing with reported energy-use at three scales: nation-wide, state-wide, and city-level. Spatially granular AllUrE data aggregated nationally show good agreement with national totals (<2% difference). The goodness-of-fit ranged from 0.78–0.95 for comparison with state-level totals, and 0.90–0.99 with city-level data for different sectors. The relatively strong alignment at all three spatial scales demonstrates the value of AllUrE-India data for modelling urban energy transitions consistent with national energy and climate goals.

## Abstract (summary)

**Table:** 

Measurement(s)	electrical energy • fossil fuel • energy use
Technology Type(s)	machine learning • statistical method
Factor Type(s)	end-use energy by activity sectors in cities
Sample Characteristic - Environment	city
Sample Characteristic - Location	India

Machine-accessible metadata file describing the reported data: 10.6084/m9.figshare.13516925

## Background & Summary

India is the third-largest energy user in the world and contributed ~7% of global anthropogenic greenhouse gas (GHG) emissions in 2018^1^. Approximately 416 million people (a 90% increase) will be added to the Indian urban population from 2018 to 2050^2^. This rapid urbanization will drive up India’s energy use and GHG emissions as almost all urban centers expand, and towns become more urban. GHG emissions per capita in India were 1.94 metric tonnes in 2018 (the 131st in the world)^1^, with an annual 6% increase over the past decade^3^. India’s urbanization thus represents an important opportunity to slow energy demand and associated carbon emissions if low-carbon city planning is undertaken as the country urbanizes. Low-carbon urbanization has implications for meeting both India’s and global commitments to the Paris Agreement on climate change^4^. To achieve low-carbon urbanization, we first need data on current energy use and carbon footprints in cities to establish a baseline for tracking progress. While the Indian government has conducted district-level surveys on various social-economic-infrastructural attributes, there is no baseline energy-use dataset for all urban areas in ways that: (a) capture key local energy-use features, (b) align with national totals, and (c) integrate social-infrastructural-urban form variables^5^. Such baseline data are essential for India and its cities to develop and evaluate low-carbon policies that align from local to state and national scales.

India has pledged to reduce GHG emissions per gross domestic output by 33%~35% by 2030 (based on 2005 levels) in its Intended Nationally Determined Contribution (INDC) under the Paris Agreement^6^. India’s INDC emphasizes transitions to clean and highly-efficient energy systems, while no numeric targets are detailed for controlling urban emissions^6^. Part of the reason is the lack of relevant data with urban-to-nation linkage. Moreover, the method to quantify urban carbon emissions is different from the national carbon inventory. Typically, countries report direct emissions from fuel combustion using a source-based accounting method, which is the foundation for spatially gridded datasets, such as EDGAR^7^ and ODIAC^8^. Both inventories focus on sources of anthropogenic GHG (e.g., fossil fuel combustion in power plants). Source-based accounting for cities can miss mitigation opportunities because urban areas provide unique avenues for deep-decarbonization through demand reduction when taking advantage of spatial efficiency (i.e., the co-location of various activities)^9–11^. Indeed, urban GHG accounting should focus on energy use by multiple users (e.g., households, manufacturing, and businesses), tracking energy use activity with trans-boundary supply of energy (e.g., power plants, refineries, etc.)^12^. The inclusion of multiple users in an urban dataset is essential to inform cross-sectoral actions (e.g., energy and material exchange across sectors), in addition to single sectoral policies (e.g., efficiency improvement and electrification). A study demonstrated Chinese urban areas supporting energy and material exchange across sectors can additionally reduce 40% of total carbon mitigated by single sectoral efficiency policies^13^. Therefore, urban areas can contribute significantly to national deep decarbonization goals. To do so effectively, both cities and nations need datasets addressing energy use across all urban areas to scale up the impact of urban actions to the national level.

Such local-to-national aligned energy use datasets covering all urban areas have been developed in the U.S^14^. and China^13,15^. More recently, efforts have been made to develop a fine-spatial scale socio-demographic and economic database in India^16^, but this dataset does not include energy use. Previous studies on energy use in India either focused on individual cities^17^ or covered individual end-use sectors across multiple cities^18–20^. These data can be used to inform energy transitions for individual or a subset of cities, or one end-use sector. However, these datasets do not address energy use in multiple sectors across all urban areas, and do not address whether the sum of modelled energy use aligns with national total energy use. Moreover, existing data are fragmented by sectors, and social-economic employment data are not often integrated with infrastructure attributes.

Our paper addresses this gap by developing and implementing a novel bottom-up plus top-down approach to quantify energy use in four end-use sectors. This bottom-up plus top-down approach modifies a method recently used to develop data for Chinese cities^13,15^. We integrate the available social-demographics, economic, infrastructural, and urban form variables based on the social-ecological-infrastructural-urban systems framework^5,21^ to develop the All Urban areas’ Energy-use (AllUrE) dataset covering all 640 Indian districts for the year 2011. This novel dataset development method can be translated to other developing countries with relatively sparse data to develop their all-cities’ energy use and carbon footprinting databases for supporting low-carbon policies and tracking progress. This dataset has many applications: exploring carbon emissions patterns across cities of different types, evaluating the impact of urbanization levels on emissions in different sectors, and quantifying collective impact of urban decarbonization strategies on national INDCs. These specific applications are beyond the scope of this paper, which focuses on describing the dataset development.

## Methods

### Method overview

The urban units in our dataset correspond to urban portions of all 640 districts specified by the Government of India, for which this AllUrE dataset provides baseline energy use consistent with the national total for the first time. We chose the year 2011 because it provided the latest publicly available population census, with detailed demographics, employment, housing conditions, residential energy use structure, and water sanitation infrastructure data covering both rural and urban areas in each district.

In the Census of India 2011, 27 states and 8 union territories representing the whole of India are divided into 640 districts. Within a district, there are towns and villages. The Census of India 2011 uses the phrase “urban area” to refer to statutory towns or census towns. Statutory towns are administrative units defined as having urban status, including Municipal Corporations, Municipalities, Cantonment Board, Notified Town Area Committee, Town Panchayat, etc.^22^. Census towns are defined by three criteria, which are (a) over 5,000 persons, (b) 75% of male workers engaging in non-agricultural activities, and (c) population density higher than 400 persons/sq. km^22^. The sum of statutory and census towns in a district is the urban area within the district. Remaining geographic areas, excluding statutory and census towns, are classified as rural areas. According to the Census of India 2011, about 31% of Indian total population lives in urban areas. Of the 640 districts, three districts have no defined urban areas (meaning no statutory or census towns are in these districts). Twenty-four districts have over 80% of their population living in defined urban areas, and nine of these 24 districts have 100% urban population (meaning these districts are the sum of statutory or census towns). While our focus was on the energy use in urban areas, the rural portion was also estimated to ensure the addition of energy use across all districts aligned with national totals.

**Fig. 1:**
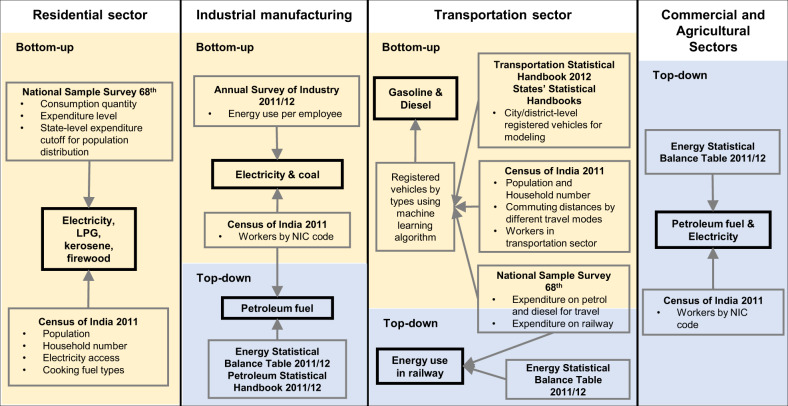
Diagram showing the data sources to estimate All Urban areas’ Energy-use across 640 districts’ urban areas in India by end-use sectors (i.e., residential, industrial, transportation, and commercial & agriculture sector).

The overarching process for estimating All Urban Areas’ Energy-use (AllUrE) across 640 districts of India is shown in Fig. 1. Energy end-use sectors included households, industrial manufacturing, transportation, and commercial and agricultural sectors. End-use energy included coal, petroleum fuel (e.g., liquefied petroleum gas (LPG), kerosene, gasoline, and diesel), electricity, and firewood (only in the residential sector). The bottom-up approach estimated energy use in households and industrial manufacturing (coal and electricity use only) based on three data sources (i.e., the Annual Survey of Industry 2012^23^, the National Sample Survey 68^th^ Round 2011/2012^24^, and the Census of India 2011^25^) that reflected local social, economic, and demographic attributes. Fuel use for on-road transportation was quantified based on the number of vehicles and their traveling distance (see Fig. 1). For activities where surveys were not available, including the commercial sector, agricultural activity, and petroleum fuel use in industrial manufacturing, we applied a top-down approach to downscale national energy use reported by the Government of India to the district level based on the number of workers in these sectors. The main data sources for the top-down estimate of energy use by sectors and fuel types included the National Energy Statistical Yearbook^26^ and Indian Petroleum and Natural Gas Statistics^27^. The modelled energy use data by end-use sectors in all urban areas were merged with publicly available social-demographics, economic, infrastructural, and urban form variables, which were primarily collected from the Census of India 2011 (see Data Records section for details).

**Fig. 2:**
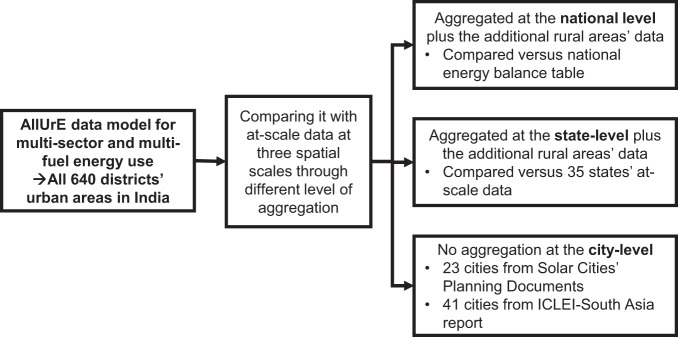
Schematic illustration of technical validation of energy use data in AllUrE-India at three spatial aggregation scales: (1) national-level aggregation; (2) state-level aggregation; (3) un-aggregated data at the city-level with aligning of appropriate boundary.

The quality of modelled energy use in AllUrE-India was evaluated at three spatial aggregation scales (i.e., national, state, and city scales) (see Fig. 2). First, the sum of energy use in AllUrE in urban and rural areas across the 640 districts nation-wide was compared against national total energy use reported in Indian statistical yearbooks. The alignment of estimated data with national totals is a key criterion, because a high alignment ensures that data can be used to quantify the carbon mitigation potential of collective city-level actions on national outcomes. Second, the sum of energy use in urban and rural areas across the 640 districts was compared against the available at-scale data for all states (27 states and 8 union territories). Lastly, the estimated urban energy use data was compared against the city-level data collected from literature (ICLEI-South Asia report provided 41 cities energy use for 2007/2008^18^) and urban planning documents (e.g., solar cities’ planning documents yielding 23 city-level comparison points). This city-level comparison reveals the effectiveness of the bottom-up approach in estimating cities’ energy use.

#### Residential sector

Energy use in the residential sector includes electricity, LPG, kerosene, and firewood used for cooking, lighting, and heating/cooling. Gasoline and diesel used for personal travel are included in the transportation sector and not included in the residential sector.

Each urban area’s residential energy use was calculated as the sum of average energy use per person within five quintiles (i.e., bottom 20%, 20–40%, 40–60%, 60–80%, and 80–100%) obtained from the National Sample Survey (NSS) multiplied by the population in these quintiles (see Eqs. 1 and 2 for electricity and LPG as examples). We differentiated between these quintile strata because they can better represent the variation of residential energy use per household^28^. For example, Nagpure *et al*. (2018) showed that per capita electricity use in the highest 20% income stratum is 5.5 times that of the lowest income stratum^29^. The effectiveness of this approach is evaluated by comparing against utility reported data at the city level (see Technical Validation).

The NSS collected 101,651 households’ data from 7,469 villages and 5,268 urban blocks across India in the 68^th^ Round (2011/12)^24^. Each surveyed household reported the amount of monthly energy use in physical quantities (e.g., kilogram, kWh, or litre). The number of households surveyed in each district was used to calculate the error in the mean by monthly expenditure strata. Both total population and the access to energy data for each district’s urban area were extracted from the Census of India 2011^30^.

The average energy use per person (e.g., *EPC*_*ele,i,j*_ and *EPC*_*ele,i,j*_ for electricity and LPG use per person, respectively) in a district’s urban area was calculated in five population strata, divided based on households’ monthly expenditure levels. Each stratum accounted for 20% of the urban district’s population and ranked from the lowest to the highest expenditure levels. The expenditure cut-offs for the population strata were summarized in the NSS Household Expenditure Report^31^ for each state’s urban and rural areas. When a district’s urban area had less than ten samples within an expenditure range, household samples from the state’s urban area with the same expenditure level were used in the estimation. The physical quantity of energy use per person in the wealthiest 20% of the population was capped at the third quantile value of this sample to remove outliers.

Total electricity use in each district’s urban area was calculated based on Eq. (1):

1 $$RE{S}_{ele,j}={\sum }_{i}PO{P}_{i,j}\ast {a}_{ele,i,j}\ast EP{C}_{ele,i,j}$$

in which *POP*_*i,j*_ is the population in each *j*^th^ district’s urban area in the *i*^*th*^ population stratum (*e.g*., bottom 20%, 20~40% of the total population) based on monthly per person expenditure; *a*_*ele,i,j*_ is the percentage of households having access to electricity in the *i*th population stratum based on expenditure levels in the *j*th district’s urban area. Households that did not have access to electricity were assumed to have lower monthly expenditure level. *EPC*_*ele,i,j*_ is the average monthly electricity consumption per person (in the unit of kWh/person) in the *j*th district’s urban area for the *i*^th^ population stratum.

Indian households can use multiple fuel types for lighting and cooking. For example, LPG, kerosene, and firewood can all be used for cooking^31^. Each surveyed household in NSS specified what their primary cooking fuel was, although the reported consumption of each type of energy was used for all end-use purposes in the households. In the Census of India 2011, each district reported the number of households using each type of primary energy for cooking. The process of calculating residential LPG use was similar to the process of modelling electricity use, with additional consideration of the proportion of households using LPG as primary cooking fuel (see Eq. 2).

2 $$RE{S}_{LPG,j}={\sum }_{i}\,PO{P}_{i,j}\ast {a}_{LPG,i,j}\ast EP{C}_{LPG,i,j}^{1st}+{\sum }_{i}\,PO{P}_{i,j}\ast {p}_{LPG,i,j}^{2nd}\ast EP{C}_{LPG,i,j}^{2nd}+{\sum }_{i}\,PO{P}_{i,j}\ast {p}_{LPG,i,j}^{3rd}\ast EP{C}_{LPG,i,j}^{3rd}$$

in which, *a*_*LPG,i,j*_ is the percentage of households using LPG as primary cooking fuel in the *i*^*th*^ population  stratum by expenditure in the *j*^*th*^ district’s urban area. The proportion of households using LPG as the primary cooking fuel was extracted from the Census of India 2011^30^. $EP{C}_{LPG,i,j}^{1st}$ (in the unit of kg LPG/person) was calculated from households whose primary cooking fuel was LPG in the *i*^th^ population stratum in the *j*^th^ district’s urban area. ${p}_{LPG,i,j}^{2nd}$ and ${p}_{LPG,i,j}^{3rd}$ are the proportion of households using LPG as the secondary fuel or tertiary fuel. When *POP*_*i,j*_ is multiplied with *a*_*LPG,I,j*_ or ${p}_{LPG,i,j}^{2nd}$ or ${p}_{LPG,i,j}^{3rd}$, the actual population in each stratum using LPG as primary, secondary, and tertiary fuel are different and represent the fact that there is multi-fuel use in India with some households using LPG as primary, while others may use LPG as secondary or tertiary fuel for which the data were derived from the NSS. $EP{C}_{LPG,i,j}^{2nd}$ and $EP{C}_{LPG,i,j}^{3rd}$ is the average monthly per person LPG used as the secondary and tertiary fuel for the *i*^th^ population stratum in the *j*^th^ district’s urban area.

The amount of kerosene consumed in households was calculated using a similar process as described above for calculating LPG use for each district’s urban area. In contrast to LPG, kerosene can be used for both cooking and lighting. Thus, the percentage of households using kerosene as the primary cooking fuel was not used when quantifying the amount of kerosene use in each urban area. It was assumed that people had equal access to kerosene.

The AllUrE-India dataset also includes the amount of firewood used in households. The population scaling-up process for firewood use followed the same steps as Eq. 2. Our model, derived entirely from the NSS data, shows that the firewood, like other fuels, varies in its usage by quintile across different districts.

#### Industrial manufacturing

Coal and electricity use in industrial manufacturing were estimated using a hybrid approach. In this hybrid approach, we calculated average energy intensity by employee for coal and electricity use for each industry sector from the national Annual Industrial Survey (ASI) dataset^23^, and multiplied it by the number of employees in each industrial sector in a district’s urban area. The ASI is an annual factory-level survey conducted by the Minister of Statistics and Programme Implementation, Government of India^23^. The location of each surveyed factory is reported at the state level, and whether the factory is in an urban area. The ASI uses the national industry classification (NIC) code released in 2008 to categorize each factory at the 5-digit level. The number of employees working in different sectors in each urban area is extracted from the Census of India 2011^25^.

The average coal and electricity use intensity by employee by industrial sectors (*Energy Intensity*_*employee,n*_ in Eq. 3) was calculated using a composite average at the 3-digit NIC code level. Aggregating all surveyed firms nationwide at the 3-digit NIC code ensured that most industrial manufacturing sectors had more than ten firms sampled. We did not separate plants in urban and rural areas, because this separation did not reduce the coefficient of variation in energy use per employee. The average coefficient of variation across 3-digit NIC level was 1.81 for surveyed plants in urban areas, which was not statistically different from both the coefficient in rural areas (1.56) and nation-wide (1.81). Coal use per employee also showed the same pattern. Therefore, we used the national average, because we had more samples across multiple industrial types. Energy use per employee was calculated by dividing all plants’ energy input with all employees in each 3-digit NIC code because the ASI samples were stratified based on 3-digit NIC code.

Data cleaning steps were conducted to remove outliers in the ASI samples when calculating energy use intensity by employee. The total number of employees in each factory was compared with the projected number of workers (as calculated using total working days and total working man-days provided in the ASI). The k-nearest neighbours algorithm was applied to replace the number of workers in factories showing internal misalignment with their own data. ASI reported both the physical quantity and monetary expenditures on coal and electricity, which was used to calculate the expenditure per physical unit of these two energy sources to affirm that the data were overall reasonable.

The Census of India 2011 reports the number of workers in each district by 3-digit NIC code with urban or rural areas separated. Workers have been categorized into the main workers (who worked more than half a year) and the marginal workers (who worked less than half a year). The ASI reports workers based on how many employees work in a factory. A weighting factor was needed to match the number of workers in these two data sources. The Economic Census 2013/14^32^ was used to calculate the weighting factors because it covered all factories in India and was used the same way to report the number of employees as in ASI. The comparison between the number of workers in the Census of India and employees in the Economic Census yielded the weighting factor for the main workers (70%) and the marginal workers (25%). After applying the weighting factors to the number of workers reported by the Census of India, we got 30,243,052 workers, which was comparable to the 30,357,249 reported in the Economic Census. The comparison of total full-time workers at the 3-digit NIC level demonstrated a goodness-of-fit of 0.82 (in Supplementary Fig. 1), indicating the weighting factors worked for different industries as well.

Equation 3 was used to calculate the electricity and coal use:

3 $$IN{D}_{k,j}={\sum }_{n=100,j}^{n=330,j}\left(Mainworker{s}_{n,j}\ast 0.7+Marginalworker{s}_{n,j}\ast 0.25\right)\times Energy\,Intensit{y}_{employee,n}$$

in which *IND*_*k,j*_ is total *k*^*th*^ energy use (purchased electricity or coal) in the *j*^*th*^ district’s urban area. And *n* refers to the *n*^th^ 3-digit NIC code in manufacturing sectors. The main and marginal workers in each district’s urban area are from Census of India 2011. And *Energy Intensity*_*employee,n*_ is the average energy use intensity by employee in the *n*^th^ industrial sector.

In contrast to coal and electricity, which were reported in physical quantity in the ASI, petroleum data did not specify the types of fuel and were only reported in monetary units in the ASI. There were many different types of petroleum products, which were not disaggregated by type, and only expenditure on all petroleum fuel was given, making it difficult to use the same approach. Therefore, petroleum fuel use in the industrial sector was downscaled from the national level to each urban area using total workers in that sector. Petroleum fuel use for industrial manufacturing at the national level was collected from the Petroleum Statistical Yearbook^27^. The number of workers by industrial sectors was extracted from the Census of India^25^.

#### Transportation sector

Estimating energy use for on-road transportation is complex and has been done in different cities and countries using different approaches. One approach collects local gasoline sales data (e.g., ICLEI-South Asia has collected cities’ gasoline data from gas stations^18^). However, this approach is too place-specific to be applied to all 640 cities and may not accurately capture refuelling events outside the city. The second approach is to calculate energy use based on the number of vehicles in a city and the use of vehicles (e.g., vehicle kilometers travelled)^33^, which is called the vehicle-activity approach. This approach can be used to inform low-carbon mobility transitions^34^ and behavioural interventions. Some US cities collect trips’ origin and destination details in travel behaviour surveys to better capture local travel demand;^35^ however this type of survey is not available for Indian cities. The third approach uses household expenditure on gasoline or diesel for personal travel to calculate fossil fuel use along with fuel price^36^. The household expenditure-based approach is only suitable to quantify fuel used for personal travel, but not for fuel used by buses or taxis due to a lack of relationship between expenditure on these modes and fuel use.

Based on data availability in India (Supplementary Table 1), we can use either the vehicle-activity approach or the household expenditure-based approach (detailed in Supplementary Part 1) to quantify fossil fuel used for personal travels. We tested the results from two approaches and found that the fossil fuel estimate of personal private mobility from the vehicle-activity approach was highly aligned with the expenditure-based approach (Supplementary Fig. 2 shows the goodness-of-fit is 0.8). Considering these two approaches’ pros and cons, we adopted the vehicle-activity approach to estimate gasoline and diesel used for on-road travel (see details below).

The vehicle activity-based approach calculated the fuel use for on-road transportation based on Eq. (4):

4 $$TR{A}_{k,j}={\sum }_{v}\frac{\left(\left(Ve{h}_{reg,v,j}\times {\mu }_{v}\right)\times VK{T}_{v}\right)}{F{E}_{v}}\times {\tau }_{k,v}$$

in which:

- *Veh*_*reg,v,j*_ is the number of registered vehicle (the *v*^th^ type) in the *j*^th^ district’s urban area. Vehicles are categorized into five types, i.e., two-wheelers, three-wheelers, cars, taxis, buses, and light-& heavy-duty vehicles. *Veh*_*reg,v,j*_ is not available across all 640 districts. We used machine learning to get this value for all districts (method is detailed below).
- In India, registered vehicles are not deregistered even after they are no longer in use^33^. The number of registered vehicles was transferred to the number of on-road vehicles by multiplying a ratio, *μ*_*v*_. This ratio, *μ*_*v*_, was extracted from literature for different vehicle types^33,37,38^ (Table 1).

**Table 1:** Annual VKT, fuel economy, and percentage of vehicles on road for estimating fuel use in on-road transportation sector by vehicle types .

	Two wheelers	Three wheelers	Cars & Jeeps	Taxis	Buses	Light- & heavy- duty vehicles
Annual VKT per year for cities with > = 6 million population (km/vehicle)^41^	12,800	43,800	12,200	12,600	49,400	37,500
Annual VKT per year for cities with <6 million population^38^ (km/vehicle)	6,750	29,500	9,000	10,000	36,400	
Fuel economy (km/L)^a^	49.9	26.9	15.2	15.2	5.8	4.3
% of vehicle in use (*μ*_*v*_)^b^	89%	87%	97%	97%	52%	52%

^a^Fuel economy of two-wheelers, three-wheelers, cars and taxis was extracted from^37,38^. Fuel economy for buses and light-& heavy-duty vehicles was extracted from^42^.

^b^Based on comparing the age structure of different vehicle types^37,38^ and the survival rate from^33^.
- *VKT*_*v*_ is vehicle kilometers travelled for the *v*^th^ type of vehicle. Prior work in India showed that the VKT was similar in smaller cities^39^, while cities with more than 6 million had a significant increase in travel demand^40^. Considering the differences between large and small cities, we used Delhi’s VKT from^41^ for bigger cities (larger than 6 million) and VKT from^38^ for smaller cities (Table 1).
- *FE*_*v*_ is fuel economy (in the unit of km/L) of the *v*^th^ vehicle type. *FE*_*v*_ was extracted from literature^37,38,42^ (Table 1).
- *τ*_*k,v*_ is the proportion of vehicle running by gasoline or diesel. This value was extracted from^43^.

This vehicle-activity based approach captures inter-urban differences in fuel use for on-road transportation, because the number of registered vehicles demonstrates the highest variation across cities, compared to both VKT and fuel economy (Supplementary Table 2). The number of registered vehicles demonstrates variation at a factor of ~thousand, introducing the largest variation across cities (Supplementary Table 2). The variation in VKT was much smaller, with maximum of 3^33,37^, and other studies have shown even more minor variation of fuel economy of vehicles. The high agreement of vehicle-based approach versus expenditure-based approach for personal mobility across cities (Supplementary Fig. 2) indicated that the vehicle-activity approach can capture the variation of local fossil fuel use for personal mobility by focusing on the parameters contributing to the largest inter-city variation.

A machine learning approach was used to model registered vehicles (*Veh*_*reg*_) for all 640 Indian districts. First, we collected the number of registered vehicles from 109 districts (in Assam^44^, Haryana^45^, Odisha^46^, and Rajasthan^47^) and 44 large cities (with more than 1 million population)^48^. Previous local surveys found that households owning cars have on average one car per household^49^. Therefore, we used the number of households owning cars from the Census of India to represent the number of registered cars. Second, using all districts’/cities’ data, we explored the relationship between the number of registered vehicles and potential exploratory variables (see Supplementary Table 3), using a linear regression algorithm. We chose variables based on the demand for traveling by model (population size, workforce size, affluence level, education level, population density, commuting distance) and local supply level (e.g., number of workers in commercial sector providing transportation services). Specifically, the predictor variables included household expenditure on travel by different modes (estimated from the NSS^24^ using a similar approach to calculate districts’ electricity use), the total number of workers (main plus marginal workers)^25^, the number of workers working in transportation sector^25^, commuting distance by different traveling modes^30^, and vehicle ownership from the Census of India 2011 (Supplementary Table 3). We applied the stepwise variable selection procedure to select the most significant variables in models for different vehicle types. The goodness-of-fit of models for different vehicle types ranges from 0.61 to 0.90 (Supplementary Table 3). For two-wheelers, motorcycle ownership is the most important variable, explaining about 85% of variation in registered two-wheelers. For three-wheelers, buses, taxis, and light-/heavy-duty vehicles, the number of cars is the most important variable, explaining 64%, 75%, 57%, and 64% of variation, respectively. The remaining variables (expenditure level, literacy rate, commuting distance etc.) together explained an additional 4% to 10% of variation. Last, these models were the foundation to conduct supervised machine learning to improve the prediction quality. These at-scale data were divided into a training dataset (80% of all cities/districts) and a testing dataset (20% of all cities/districts). We ran algorithms using tenfold cross-validation in the training dataset. The trained model was used to predict the number of vehicles across all districts’ urban and rural areas. The predicted number of registered vehicles was used as a weighting factor to allocate the state-level registered vehicles to each district’s urban area.

***Diesel and electricity used in the railway sector*** were downscaled from the national total reported by the Government of India^26^ based on households’ expenditure on railway travel. The expenditure on the railway at the district level was calculated from NSS^24^ based on households’ monthly expenditure level.

#### Remaining sectors: commercial activities, agricultural sector, and informal businesses

The top-down approach was applied to estimate energy use in the remaining end-use sectors that could not be calculated using the bottom-up approach. These end-use sectors included the commercial activities (e.g., retail, hotels, etc.), agricultural activities, and households having informal businesses (e.g., selling street food). Petroleum and electricity use in these sectors were downscaled from the national statistics to each district’s urban area based on the number of workers, with the assumption of similar energy use per employee in the same activity sectors. The number of workers was extracted from the Census of India 2011^25^, and national total energy use in these sectors was extracted from the National Energy Balance Table^26^.

#### Data sources for energy use reported at-scale across the national, state, and local levels

***National total*** energy use in different end-use sectors was extracted from the energy balance table from National Energy Statistical Handbook 2013^26^. The petroleum fuel in the national energy balance table only included LPG, kerosene, diesel, and heavy fuel oil. The amount of petroleum used in different end-use sectors was collected from the Petroleum and Natural Gas Statistical Handbook^27^. In addition, about 85% of diesel use was marked as “end-use not specified” in both the national energy balance table and the petroleum handbook. This research reallocated the amount of diesel use to different vehicle types using data collected by the Petroleum Planning and Analysis Cell^43^.

***State-wide*** electricity in residential and industrial sectors, and gasoline and diesel use for on-road transportation data, were collected for comparisons at the state level. Both industrial and residential electricity use data at the state-level were collected from TERI Energy & Environment Data Diary and Yearbook^50^. The state-wide total gasoline and diesel use (for transportation and non-transportation purposes) were provided in the Petroleum & Natural Gas Statistical Handbook^27^. The total state-wide gasoline and diesel use for on-road transportation were extracted based on the regional gasoline and diesel use structure detailed in the All India Study on Sectoral Demand of Diesel and Petrol^43^.

***City-level energy use*** data are sparse and not available for a large number of cities over time. In this analysis, city-level energy use data were extracted from Solar City Master plans and ICLEI-South Asia’s report on GHG patterns of south Asian cities^18^. Fuel use in different end-use sectors was extracted from 23 cities’ solar city master plans. ICLEI-South Asia reported fossil fuel use by different economic sectors for the year 2007/2008 in 41 cities^18^, while there is no such report available for 2011. The reported energy use may be at the municipal corporation level, which can differ from the boundary of a city and a district’s urban area. To ensure the same boundary of reported and estimated energy use, the same approaches to evaluate energy use at the urban areas were applied to cities using social-economic data from the Census of India 2011^25,30^.

## Data Records

Because infrastructure use in cities is shaped by social-economic factors (e.g., total population and employment structure), ecological factors, infrastructure access, and urban form (e.g., population density)^5,51^, the inclusion of these non-energy use variables can help to investigate the drivers of energy use. Variables in the AllUrE-India dataset are therefore organized based on the social-ecological-urban-infrastructure (SEUI) system framework^5,21^ (Table 2).

**Table 2:** Overview of the data structure in All Urban Areas’ Energy-use across 640 Indian districts dataset providing selected examples of variables based on the Social-Economic-Infrastructural-Urban form (SEIU) framework^5,9^.

All Urban areas’ Energy-use (AllUrE) across 640 Indian Districts for 2011	
SEIU categories	Examples of variables
Social demographics	• Total population
	• Total population by gender
	• Number of households
	• Infrastructural access
	• Electricity
	• Cooking fuels by types
	• Treated water
	• Sanitation
Economic feature	• Number of workers in different economic sectors
Infrastructure use	• Energy use by end-use sector and by fuel types
	• Residential sector
	• Industrial manufacturing
	• Transportation
	• Commercial sector
Urban form	• Population density

 In the dataset, variables are categorized into five sections: social-demographic variables (including infrastructure access variables), economic activities, energy use in four end-use sectors, and urban form (see Table 2). The ecological variables are not included in the AllUrE-India dataset due to the lack of broad coverage of such city-scale data in India. The Census of India 2011 reported socio-demographics (e.g., population, household number, age of population etc.) and households’ access to infrastructural services (e.g., access to electricity, water, and sanitation) information at the district level with urban and rural areas separated. These non-energy use variables in AllUrE-India were all extracted from the Census of India 2011^25,30^ and merged with urban energy use data. An Excel file including the AllUrE-India 2011 datafile (tab name “AllUrE_20210104”) and codebook (tab name “codebook”) can be downloaded from 10.6084/m9.figshare.12331283.v3^52^.

## Technical Validation

The quality of energy use data in the AllUrE-India dataset and the effectiveness of the bottom-up approach have been evaluated by comparing the sum of energy use from AllUrE-India and the estimates of rural areas’ energy use with the national total reported by the Government of India. The same aggregation has been done at the state level to investigate the state-level alignment. A high alignment with at-scale national and state totals ensures that the AllUrE-India dataset can be used to evaluate the energy-saving and carbon mitigation potential of collective urban actions at the national or state level. We compared the effectiveness of a bottom-up approach with respect to the city-level data when such data were available.

### National level alignment

Table 3 demonstrates total national energy use aggregated from AllUrE-India data modelled for 640 districts versus the at-scale national energy use reported by the government (Government of India, GoI) in different end-use sectors by fuel types. The total estimated national end-use energy is about 1.7% less than the national total reported by GoI. The differences in aggregated national energy use by types ranges from -11.6% to 6.2%.

**Table 3:** National-scale validation: comparing energy use estimate aggregated nationally across 640 districts versus national total reported by the Government of India (GoI) in (a) industrial manufacturing, (b) residential sector, (c) on-road transportation. The comparison of total end-use energy by types was shown in part (d).

Fuel type by end-use sector	National aggregation from AllUrE-India for 640 districts	At-scale national total energy use from GoI	Difference (GoI’s data as the base)
**a. Industrial manufacturing**			
Coal and lignite (kilo tonne)	221,279	238,538	−7.2%
Electricity (GWh)	351,216	331,158	6.1%
**b. Residential sector**			
LPG (kilo tonne)	**10,693**	12,365	−13.5%
Kerosene (kilo tonne)	7,909	7,922	−0.2%
Electricity (GWh)*	197,606	170,034	16.2%
**c. On-road transportation**			
Gasoline (kilo tonne)	14,479	14,932	−3.0%
Diesel (kilo tonne)**	44,796	42,911	4.4%
**d. Sum of national total electricity and fossil fuel end-use**			
Coal and lignite (kilo tonne)***	221,279	238,538	−7.2%
LPG (kilo tonne)	12,693	14,364	−11.6%
Kerosene (kilo tonne)	8,093	8,106	−0.2%
Gasoline (kilo tonne)	14,573	14,932	−2.6%
Diesel (kilo tonne)	66,086	64,742	2.1%
Heavy fuel oil (kilo tonne)	6,455	6,455	0.0%
Electricity (GWh)	818,232	770,603	6.2%
Sum of energy use all above in the unit of Petajoule (PJ)	12,799	13,026	−1.7%

Note:

*In total coal use, coal used for generating electricity is not included to avoid double counting.

**Residential electricity use is higher than officially reported, because households may report the amount of electricity use for informal commercial activities.

***Diesel use for aviation is not included.

### State-level data comparison

**Fig. 3:**
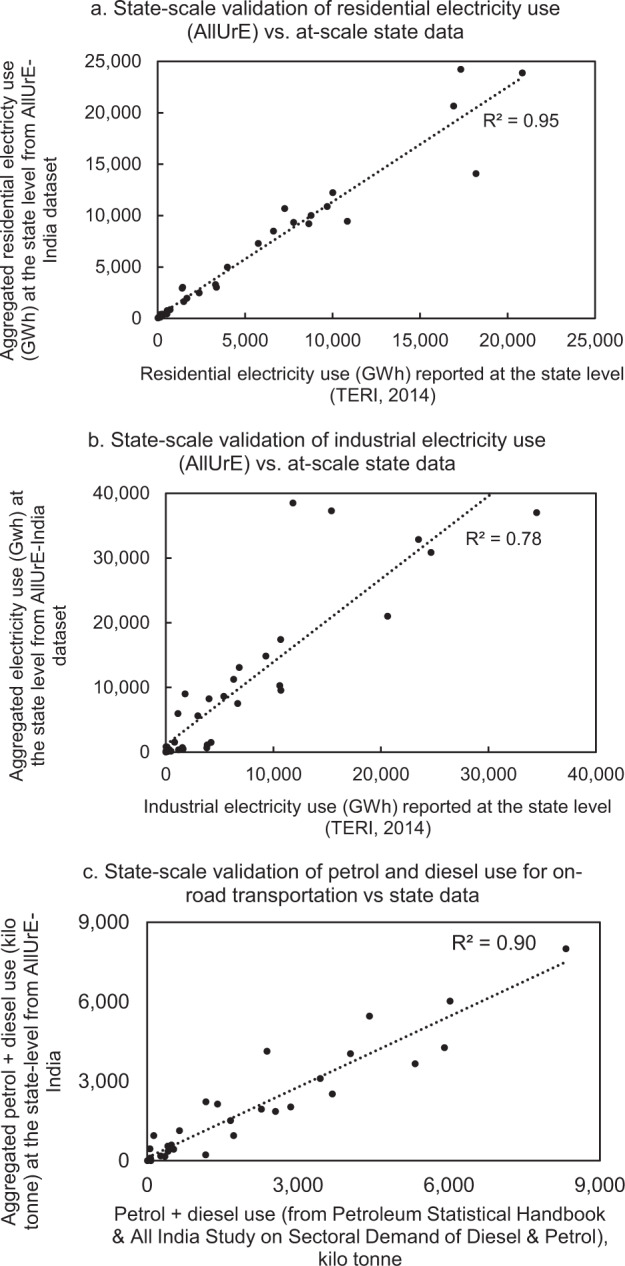
State-scale validation comparing energy use from AllUrE-India dataset in (**a**) residential sector, (**b**) industrial manufacturing, and (**c**) on-road transportation versus energy use reported at the state level.

Energy use aggregated from AllUrE-India plus the estimated rural energy use for 35 states is compared against at-scale state industrial and residential electricity use, as well as gasoline and diesel use for on-road transportation. The goodness-of-fit (*R*^2^) for electricity is 0.95 and 0.78 in the residential sector (Fig. 3a) and the industrial sector (Fig. 3b), respectively. The goodness-of-fit (*R*^2^) for gasoline and diesel use is 0.90 at the state level (Fig. 3c). These comparisons demonstrate that the estimated energy total at the state level had a high alignment with actual energy use at the state level.

### City-level comparison

**Fig. 4:**
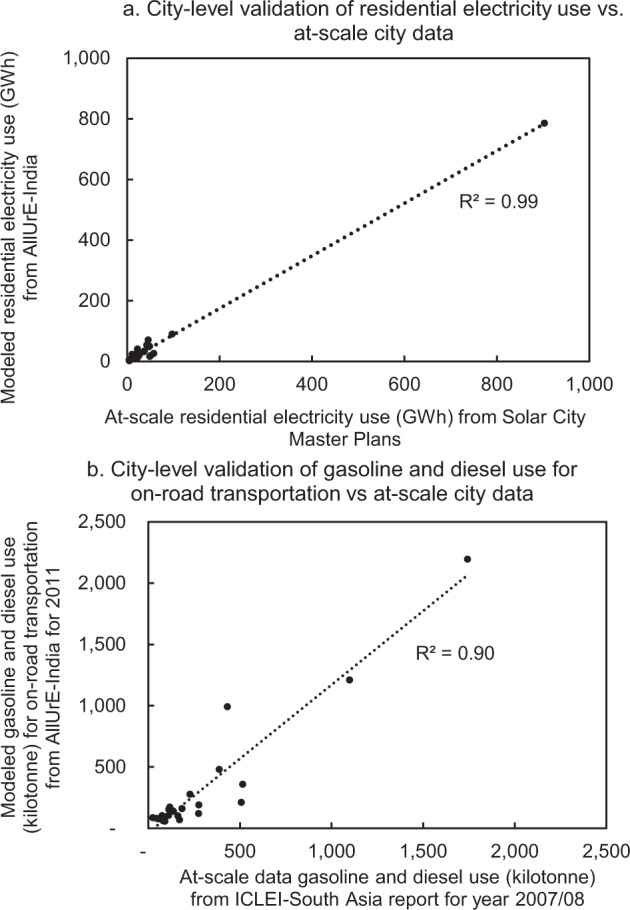
City-scale validation: (**a**) compare modelled residential electricity use in AllUrE-India versus at-scale city-level electricity use from Solar City Master Plan documents; (**b**) compare modelled gasoline and diesel use for on-road transportation in AllUrE-India versus at-scale city-level data reported by ICLEI-South Asia.

City-level energy use data are sparse and do not have broad coverage for a large number of cities during the same period. At-scale gasoline and electricity-use data reported by utilities can themselves have uncertainty. For example, non-technical electricity loss can be as high as 20% in India^53^. When comparing AllUrE against the available at-scale data, it is found that the goodness-of-fit for electricity-use at the city-level is 0.99 (Fig. 4a), and is 0.90 for gasoline and diesel use for on-road transport at the city level (Fig. 4b). The normalized root-mean-square error is 3% and 44% for electricity-use and on-road fuel use, respectively.

The total electricity use derived from the NSS data fits well with utility-reported energy use data (at-scale), demonstrating the effectiveness of the bottom-up methodology (Fig. 4a). Because we differentiated household energy use by expenditure quintile, the AllUrE dataset can represent not only the aggregated total, but also the inequality in residential energy use, which is highly skewed to higher income populations.

The at-scale gasoline and diesel use data are for the period of 2007-2008, while the estimated transportation fuel use is for the year 2011. Furthermore, the estimated diesel and gasoline use based on local registered vehicle number is used for both local and long-distance travel. In comparison, diesel and gasoline use reported at the city-scale is based on the sales data from local gas stations^18^, meaning that amount of diesel is sold locally. Thus, different methods of estimating energy use for on-road transportation explain the difference between the at-scale and estimated data.

The validation across spatial scales demonstrates a new aspect of urban data science, where we are gathering data across all urban areas consistent with national and provincial/state levels with the focus on energy use rather than the location of fuel combustion.

## Usage Notes

Overall, the database is novel in representing energy use in multiple sectors based on unique features of each urban district, while also aligning total energy use by different fuel types across spatial scales (city, state, and national). Specifically:

- Residential energy use captures both the population and inequality in access to energy, and consumption of energy in each district, using census data for population and access, along with consumer survey data for inequality in consumption.
- Industrial energy use mostly follows the number of industrial manufacturing workers by sector in each city from census data with sectoral energy use intensity derived from the Annual Industrial Survey. Commercial energy use reflects the number of workers in commercial sectors in each city. Thus, the employment activity in each district is reflected in energy use.
- Transportation energy use is calculated using modelled registered vehicles at the city level, which in turn is found to be highly correlated with local social-economic parameters in each district (e.g., automobile ownership, education, per capita expenditures, literacy), along with miles travelled represented for city-types in India (larger than 6 million or smaller than 6 million population).

Urban areas in this dataset were the aggregated administrative towns and municipalities defined by the census in each district. They covered all census defined cities, which are not equal to the total of urban agglomerations defined by remote sensing data.

The use of firewood has not been compared against any at-scale data, because biomass use is not reported in governmentally released statistical yearbooks. The effectiveness of this bottom-up method in the residential sector has been demonstrated when evaluating electricity and fossil fuel use. The uncertainty in estimated firewood was assumed to be the same as the electricity estimate in the residential sector.

City-specific freight data are very challenging to collect. In US cities, prior work has used input-output economic data to evaluate long-distance freight costs^54^. In India, limited data are available for long-distance freights at the city level^55,56^. There are data only for the 20 largest metro areas in India^55,56^. In general, the bigger the city, the more freight goes into the city^57^. In the analysis, we used 153 districts’ at-scale reported trucks to build a model to predict the number of trucks in the rest of the cities, in which the number of workers (another metric for city size) demonstrated significant impacts on the number of registered trucks. The trucks’ VKT and fuel economy were extracted from previous studies analysing energy use and air pollutant emissions^33^. Although the energy use for freight is not combustion location-specific, fuel use for freights indicates how much energy is needed to transport goods in and out of the city. On-road freight accounts for 6.9% of national total end-use energy. Although there may be uncertainty at the city level, the energy balance at the national level is not influenced.

End-use energy in commercial and agricultural sectors was downscaled using the number of employees as a proxy. The top-down approach used for these two sectors in this research only ensures the alignment with the national total, while local activity features are not captured. Thus, the use of data to inform local energy transitions in these two sectors is limited. In the future, new approaches can be used to quantify commercial buildings’ energy use. For example, an alternative approach is to quantify energy use based on energy use intensity per floor area instead of energy use per employee. An alternative approach to estimate energy use in agricultural sectors is using the amount of food production and energy used per unit of food. Future research can explore new data analytical techniques and new data sources to support the development of these alternative bottom-up approaches to quantify energy use in these two end-use sectors.

Nowadays, the emerging urban data revolution provides more data and analytical approaches to model urban energy use that are being tested in other countries, which can be translated in the future to India and other countries. Table 4 presents the representative data sources and methods to generate such data at the urban level. Translating this to India will require more on-the-ground data and fieldwork, combining satellite data with cell phone data and on-the-ground surveys. In addition to mining emerging data, local and national governments can conduct surveys, such as travel demand surveys and commercial buildings energy use surveys, to develop urban energy use databases.

**Table 4:** Emerging data sources and methods that can be used for developing all urban areas’ energy use databases.

End-use sector	New data sources	New method for data estimation	Examples
Households	Street imagery data Phone tower data	Artificial intelligence or deep-learning approaches to process street imagery data to extract social-demographic, income data, rooftop solar PV.	• Suel *et al*. (2019) processed street imagery to extract detailed social-demographic data, i.e. income, education, unemployment, housing, living environment, health and crime^58^. • Gebru *et al*. (2017) analyzed Google Street View images of cars to extract neighborhoods’ socioeconomic attributes, e.g. income, race, education, and voting patterns^59^. • Yu *et al*. (2018) processed imagery data to get all roof-top solar PV panels across the US^60^. • Barbour *et al*. (2020) applied phone tower data to estimate building occupancy for estimating energy use^61^.
Industrial manufacturing	Satellite data, imagery data, global production data and production index to extract fossil fuel use.	Satellite data using short-lived air pollutants and local survey data to identify plants. Using the production data to allocate energy use to fine temporal scale.	• Van Damme *et al*. (2018) identified the ammonia point sources in industrial and agricultural sectors using satellite NH3 measurement data^62^. • Liu *et al*. (2020) applied industrial production data to allocate fossil fuel associated CO_2_ emissions to daily scale^63^.
Transportation	GPS data for traffic congestion, etc. Phone tower data	Using big data, e.g., machine learning, and artificial intelligence techniques	• tomtom.com provides traffic congestion index for 416 cities across the globe^63^. • streetlightdata.com employs high spatial-temporal solution data and survey data to extract travel demand.

In this research, a novel bottom-up plus top-down approach is developed and adopted to assemble the AllUrE-India database for the year 2011. The database represents energy use in multiple sectors across all 640 urban districts of India, leveraging a number of surveys and machine learning models, representing both the unique features of individual urban areas, while also aligning total energy use by fuel types across city, state, and national totals. The method developed in this research is applicable to other countries with survey data, such as households and industrial/commercial energy use surveys to develop their all-cities’ energy use and carbon footprinting databases.

## Supplementary information

Supplementary Information

## Data Availability

Data were processed in R version 3.5. and code was written in RStudio 1.1.463. The sample code to calculate residential electricity and LPG use using the National Sample Survey can be downloaded from^52^.
