# Supplementary material for: All urban areas’ energy use data across 640 districts in India for the year 2011
Source: Sci Data. 2021 Apr 12;8:104. doi: 10.1038/s41597-021-00853-7 (PMC8041814; doi:10.1038/s41597-021-00853-7)
Supplement: Supplementary file 1 — Supplementary Information [file 41597_2021_853_MOESM1_ESM.docx]

**Supplementary Information for**

### All urban areas’ energy use data across 640 districts in India for the year 2011

Kangkang Tong^1^, Ajay Singh Nagpure^2^, Anu Ramaswami^1,2,3,4,*^

**Affiliations**

1. Department of Civil and Environmental Engineering, Princeton University, Princeton, NJ, USA

2. Hubert H. Humphrey School of Public Affairs, University of Minnesota-Twin Cities, Minneapolis, MN, USA

3. M.S. Chadha Center for Global India, Princeton University, Princeton, NJ, USA

4. High Meadows Environmental Institute, Princeton University, Princeton, NJ, USA

*corresponding author(s): Anu Ramaswami ([anu.ramaswami@princeton.edu](mailto:anu.ramaswami@princeton.edu))

**Table of contents**

**Supplementary Part 1**. Comparing estimated fossil fuel use for personal private mobility from household expenditure-based and vehicle-activity approach ……………………………………………………………………………(1)

**Supplementary Table 1**. Data sources to estimate fuel use for on-road transportation in India…………………………………………………………………………………………………………………………………………………(2)

**Supplementary Table 2**. The variation of fossil fuel use for on-road transportation at inter-urban scale primarily influenced by the number of vehicles….……………………………….…………………………………………….(3)

**Supplementary Table 3**. Linear regression results to model the number of registered vehicle by different types using all at-scale districts/cities’ data….…..……………………………………………………………………………….(4)

**Supplementary Figure 1**. Comparing number of workers from the Census of India (with weighting factors) (shown at x-axis) vs. Economic Census (shown at y-axis) at the 3-digit NIC-code level.………..(5)

**Supplementary Figure 2**. Comparing estimated fossil fuel use for private mobility based on vehicle-based approaches (x-axis) versus household expenditure-based approach (y-axis)………………………….(6)

# **Supplementary Part 1: Comparing estimated fossil fuel use for personal private mobility from household expenditure-based and vehicle-activity approach**

Supplementary Table 1 lists key data sources to model fossil fuel use for on-road transportation in India at the district level. Based on data availability, we had two approaches to quantify fossil fuel used for personal private mobility, and one approach for freights/goods travels.

For personal private mobility, the first approach is to quantify fuel based on household expenditure on petrol and diesel for travel. This approach calculated total petrol and diesel use for personal mobility in a city by dividing cities’ total expenditure on petrol or diesel for personal mobility by the fuel price. Total expenditure on petrol and diesel was estimated based on monthly expenditure quintile in urban areas. The average expenditure on petrol and diesel in each quintile was calculated from NSS data, which was multiplied with the population size to get each quintile's total in a city. The price of petrol and diesel was assumed to be the same across cities, because no price dataset was available across all districts and the variation in fuel prices was small across cities (e.g. 81.06 in Delhi to 87.74 rupees per liter in Mumbai for gasoline, and 70.46 to 76.86 rupees per liter for diesel^1^). The drawback of this approach is that it can only be used for personal travel, not for buses and taxi, because of the unknown relationship between expenditure and fuel use for taxis and buses. Therefore, this approach was used as a comparison with the vehicle activity-based approach.

The comparison between expenditure-based versus vehicle activity approach results is shown in Supplementary Figure 1. Due to the more considerable variation of fossil fuel use, we used a natural logarithm transformation for estimated fossil fuel use. This figure demonstrates a very high goodness-of-fit (*R^2^* = 0.80) between the two approaches. The expenditure-based approach can be applied to compare the vehicle-based approach's estimate to understand whether the vehicle-based approach captures local traveling demand across cities. We are confident in the vehicle activity-based approach's performance to reflect local fossil fuel use, although the estimate of VKT and fuel economy are the same. In future research, new approaches to reflect more local fuel economy and VKT by types can improve the estimate further, where we need new data sources and analytical technology.

**Supplementary Table 1**. Data sources to estimate fuel use for on-road transportation in India

| Data sources | What data are available |
| --- | --- |
| **National Sample Survey 2010/11**  (covering all states districts with urban and rural areas separated, but 26 districts were not covered) | - Household expenditure in the unit of rupees on petrol and diesel for traveling - Household expenditure on buses, taxis - Household asset ownership |
| **Census of India 2011**  (covering all states districts with urban and rural areas separated) | - Number of households owning cars, motorcycles - Person-distance of commuters by different type of vehicles (i.e., cars, two-wheelers, three-wheelers, buses, taxis) |
| **Consumer Pyramid 2013/14**  (covering 260 districts’ urban areas, and 300+ districts’ rural areas) | - Household assets ownership - Household income |
| **On Road Transportation Statistics (**cities with 1+ million population)  **State-level Statistical Yearbook** (state total) | - Registered vehicles by types in cities with more than 1 million population - Three states reported the registered vehicles |

**Supplementary Table 2**. The variation of fossil fuel use for on-road transportation at inter-urban scale primarily influenced by the number of vehicles

| **Type of vehicles** | **Fuel economy (km/liter) range^1^** | **Annual VKT (km) range^2^** | **The range of the number of registered vehicles ^3^** |
| --- | --- | --- | --- |
| Two-wheelers | 48 ~ 53.2 | 3500 ~ 10,000 | 501 ~ 4,661,714 |
| Three-wheelers | 22.5 ~ 30 | 19,000 ~ 44,000 | 1 ~ 222,127 |
| Cars | 12.5 ~ 16.4 (for gasoline)  14.0 ~ 15.8 (for diesel) | 8,500 ~ 12,600 | 95 ~ 2,240,717 |
| Buses (including taxis) | 2.9 ~ 3.3 (for diesel)  40 ~ 48 km/kg (for CNG) | 16,000 ~ 49,500 | 0 ~ 171,850 |
| Light- & heavy- duty vehicle | 3.3 ~ 6.6 | 30,000 ~ 68,000 | 0 ~ 135,240 |

Notes:

1. From ^37,38,41,44^

2. From ^33,41^

3. From 153 district and cities which reported the number of registered vehicles by types. The lowest value is reported by the District of Deogarh in the State of Odisha

**Supplementary Table 3**. Linear regression results to model the number of registered vehicle by different types using all at-scale districts/cities’ data

| **Response variables** | **Predictor variables** | **Adjusted *R^2^*** |
| --- | --- | --- |
| Two-wheelers (at natural logarithm scale) | ln(# of registered cars), coef. = 0.403 (***)  ln(# of HHs owning motorcycles), coef. = 1.238 (***)  ln(# of workers), coef. = - 0.584 (***)  ln(monthly expenditure per person), coef. = -0.638 (**)  ln(commuting distance by two-wheeler per worker), coef. = 0.534 (*)  ln(female literacy rate), coef. = -0.547 (*) | 0.905 |
| Three-wheelers (at natural logarithm scale) | ln(# of registered cars), coef. = 0.620 (***)  ln(# of workers in urban passenger transportation), coef. = 0.592 (***)  ln(population density), coef. = 0.289 (**)  ln(commuting distance by three-wheeler per worker), coef. = -0.695 (.)  ln(total household expenditure on diesel for travel), coef. = -0.298 (*)  ln(monthly expenditure per person), coef. = 0.364 | 0.741 |
| Taxis (at natural logarithm scale) | ln(# of registered cars), coef. = 0.752 (***)  ln(# of workers in urban passenger transportation), coef. = 0.390 (***)  ln(total household expenditure on petrol for travel), coef. = - 0.63 (***)  ln(total household expenditure on taxi), coef. = 0.558 (***) | 0.613 |
| Buses (at natural logarithm scale) | ln(# of registered cars), coef. = 0.683 (***)  ln(# of workers in urban passenger transportation), coef. = 0.160  ln(percentage of HH having mobile phones), coef. = 0.584  ln(population density), coef. = 0.159  ln(female literacy rate), coef. = - 0.909 (**)  ln(total household expenditure on buses), coef. = 0.238 (***)  ln(monthly expenditure per person), coef. = -0.987 (*) | 0.754 |
| Light-/heavy-duty vehicles | ln(# of registered cars), coef. = 0.558 (***)  ln(# of workers in the freight transportation sector), coef. = 0.446 (***)  ln(population density), coef. = 0.142 (*)  ln(monthly expenditure per person), coef. = -1.210 (**)  ln(percent of HHs having mobile phones), coef. = 1.617 (***) | 0.727 |


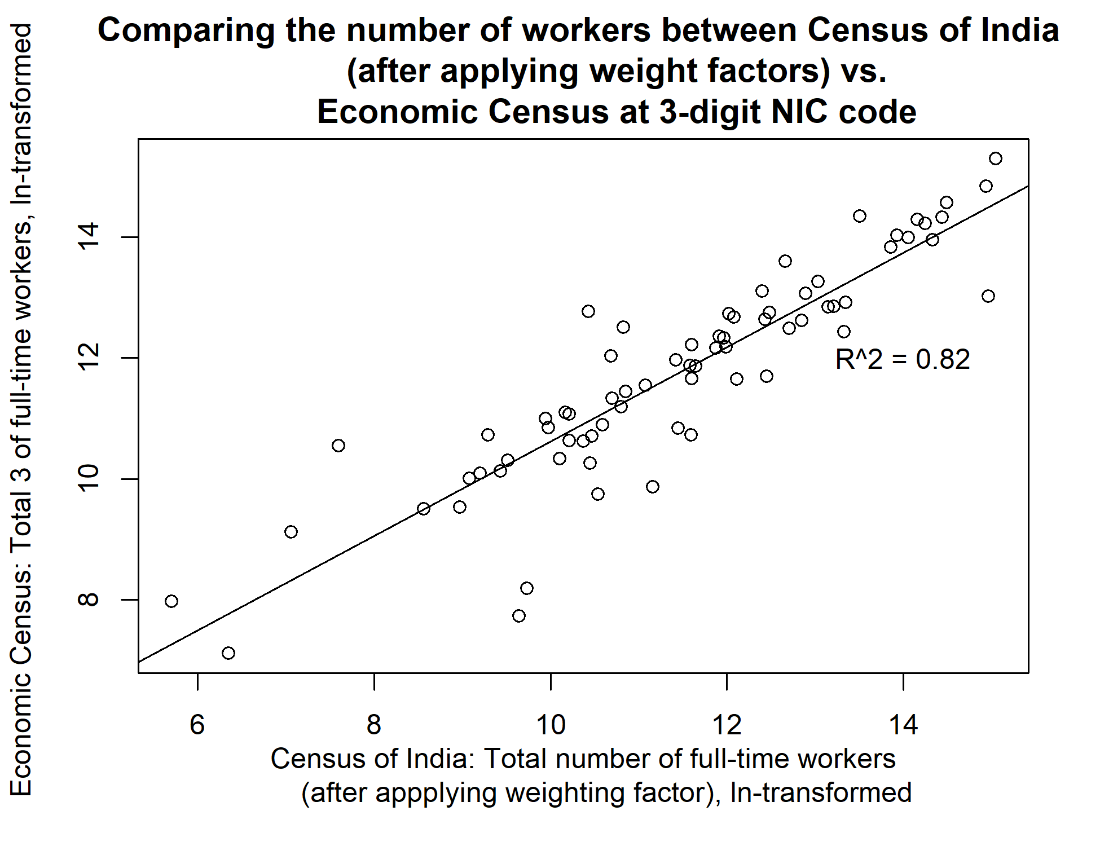

**Supplementary Figure 1**. Comparing number of workers from the Census of India (with weighting factors) (shown at x-axis) vs. Economic Census (shown at y-axis) at the 3-digit NIC-code level


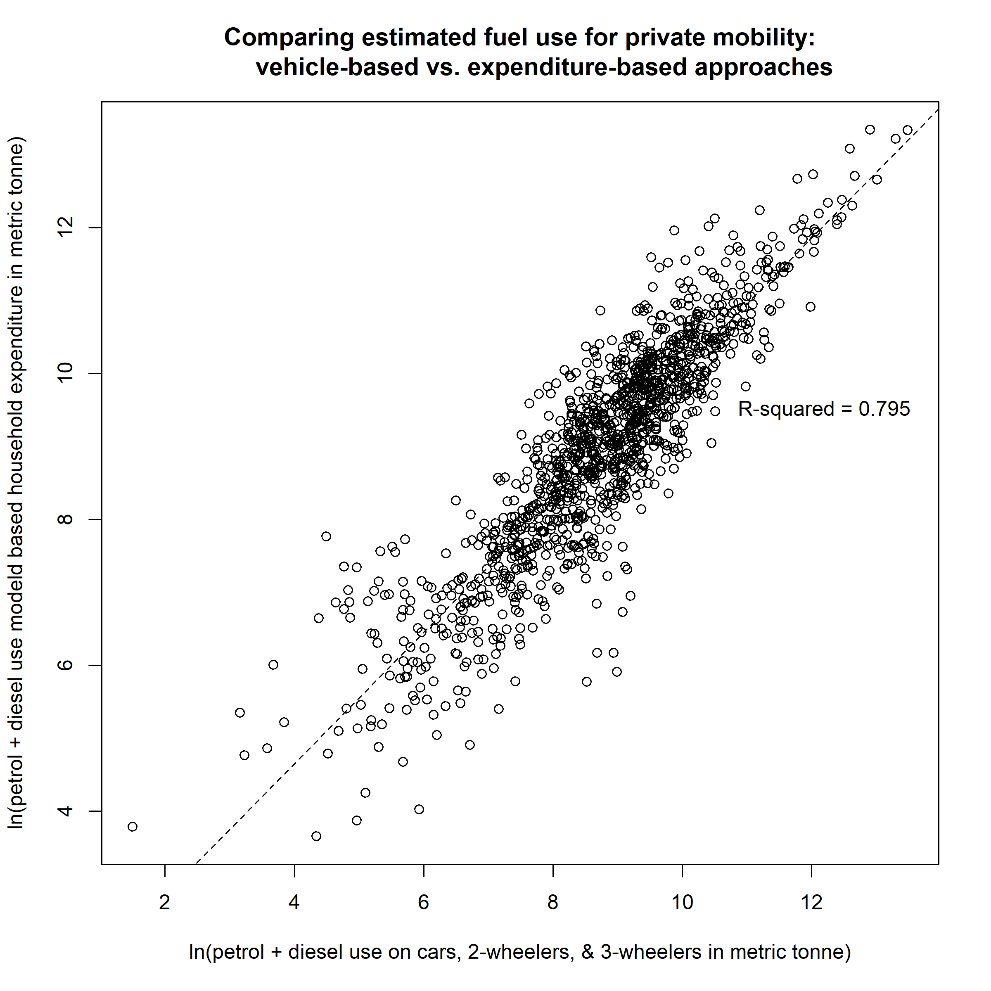


**Supplementary Figure 2**. Comparing estimated fossil fuel use for private mobility based on vehicle-based approaches (x-axis) versus household expenditure-based approach (y-axis)

References:

1 Noronha, P. *No Change In Petrol, Diesel Prices On Thursday*, <https://www.ndtv.com/business/petrol-price-today-petrol-diesel-prices-see-no-change-on-thursday-october-29-check-latest-rates-here-2317271> (2020).

2 Ministry of Urban Development. Study on Traffic and Transportation Policies And Strategies in Urban Areas in India. (2008).

3 IIHS. Urban Transport in India Challenges and Recommendations. (Indian Institute for Human Settlements, 2015).

4 Goel, R., Guttikunda, S. K., Mohan, D. & Tiwari, G. Benchmarking vehicle and passenger travel characteristics in Delhi for on-road emissions analysis. *Travel Behav. Soc.* **2**, 88-101, <https://doi.org/10.1016/j.tbs.2014.10.001> (2015).

5 Goel, R., Mohan, D., Guttikunda, S. K. & Tiwari, G. Assessment of motor vehicle use characteristics in three Indian cities. *Transp. Res. D Transp. Environ.* **44**, 254-265, <https://doi.org/10.1016/j.trd.2015.05.006> (2016).

6 Mohan, D., Goel, R. & Tiwari, G. Assessment of Motor Vehicle Use Characteristics in Three Indian Cities. (2014).

7 International Council on Clean Transportation. Fuel Consumption Standards for Heavy-duty Vehicles in India. (2017).

8 Pandey, A. & Venkataraman, C. Estimating emissions from the Indian transport sector with on-road fleet composition and traffic volume. *Atmos. Environ.* **98**, 123-133, <https://doi.org/10.1016/j.atmosenv.2014.08.039> (2014).
